# Clinical Characteristics and Treatment Outcomes of Patients with Low- and High-Concentration Isoniazid-Monoresistant Tuberculosis

**DOI:** 10.1371/journal.pone.0086316

**Published:** 2014-01-22

**Authors:** Tsai-Yu Wang, Shu-Min Lin, Shian-Sen Shie, Pai-Chien Chou, Chien-Da Huang, Fu-Tsai Chung, Chih-His Kuo, Po-Jui Chang, Han-Pin Kuo

**Affiliations:** 1 Department of Thoracic Medicine, Chang Gung Memorial Hospital, Chang Gung University, School of Medicine, Taipei, Taiwan; 2 Division of Infectious Diseases, Department of Medicine, Chang Gung Memorial Hospital, Chang Gung University, School of Medicine, Taipei, Taiwan; Barcelona University Hospital, Spain

## Abstract

**Background:**

Isoniazid (INH) resistance is now the most common type of tuberculosis (TB) infection resistance worldwide. The aim of this study was to evaluate the clinical characteristics and treatment outcomes of patients with low- and high-concentration INH-monoresistant TB.

**Methods:**

One hundred and thirty-four patients with culture-confirmed INH-monoresistant TB during 2006 January to 2007 December were retrospectively enrolled. INH resistance was classified as either low-concentration or high-concentration resistance according to the critical concentrations of 0.2 **µ**g/mL or 1 **µ**g/mL of INH, respectively. The patients’ clinical outcomes, treatment regimens, and treatment duration were analyzed.

**Results:**

The treatment success rates between low- and high-concentration INH-resistant TB were similar (81.8% vs. 86.7%). The treatment regimens and treatment duration were similar between both groups. Only a minor percentage of the patients in both groups received 6-month treatment regimens (low vs. high concentration resistance, 9.1% vs. 13.3%; respectively, p = 0.447) The most common reason for treatment duration longer than 6 months was pyrazinamide given for less than 6 months, followed by a delay in clinical response to treatment. Multivariable analysis showed that prior tuberculosis treatment (Odds ratio, 2.82, 95% C.I., 1.02–7.77, p = 0.045) was the only independent risk factor for unsuccessful treatment outcome.

**Conclusion:**

Different levels of INH resistance did not affect the treatment outcomes of patients with INH-monoresistant tuberculosis. Prolonged Rifampin-containing regimens may achieve those good outcomes in patients with low- and high-concentration INH-monoresistant TB.

## Introduction

Tuberculosis (TB) is the leading infectious cause of death worldwide, with 9 million new cases and nearly 2 million deaths annually [1]. Recent global surveys have reported that drug-resistant TB exists in every location [2]. Compounding the challenges of an already lengthy and complicated treatment course, the World Health Organization reported a trend toward an increasing number of cases of drug-resistant TB [3]. Isoniazid (INH) is an important first-line agent for the treatment of TB because of its potent early bactericidal activity. However, resistance to INH is very common, with a prevalence rate of 28% among previously treated cases and 10% among new cases [4]. Due to the increasing number of INH-resistant tuberculosis cases, the effect of such resistance on treatment outcomes is of particular interest.

Recent large-scale cohort studies have demonstrated that INH monoresistant TB did not decline in recent years despite the downward trends observed in overall TB cases [4], [5]. The treatment successful rates were similar in patients with INH-monoresistant and susceptible TB, however, patients with INH-monoresistant TB required longer treatment periods than those with INH-susceptible TB [4], [5]. Previous studies have reported a low rate of treatment failure (2%) for INH-resistant strains treated with an initial regimen of 4 to 5 drugs containing rifampin for at least 6 months [6]. Therefore, the American Thoracic Society (ATS), Centers for Disease Control and Prevention (CDC), and Infectious Diseases Society of America (IDSA) issued guidelines recommending treatment with a standard 4-drug regimen (INH, rifampin, pyrazinamide, and ethambutol) for 6 months, with discontinuation of INH after the results of drug susceptibility tests are known [7]. A 6-month short-course regimen of first-line drugs including INH, rifampin, pyrazinamide, and ethambutol has been reported to be as effective in drug-susceptible patients as in INH-monoresistant patients. [8] However, emerging evidence suggests that duration of treatment for longer than 6 months is required in more than half of the patients with INH-monoresistant TB [4]. In addition, highly heterogeneous treatment regimens for INH-monoresistant TB have been reported in many reports [4], [9]–[11]. Therefore, further studies are needed to evaluate the efficacy of the current treatment regimens and outcomes for INH-monoresistant TB.

INH resistance is classified as either low- or high-concentration resistance according to the critical concentrations of 0.2 **µ**g/mL or 1 **µ**g/mL of INH, respectively. According to previous studies, different genetic mutations are responsible for low- and high-concentration INH resistance [10], and they can therefore be thought of as two distinct entities. However, comprehensive studies are still lacking to address the differences between patients with low-and high-concentration monoresistant TB in terms of baseline characteristics, treatment regimen, treatment-related adverse effects, and outcomes. In addition, the *in vitro* sensitivity tests show that INH can inhibit the growth of low-concentration INH monoresistant *Mycobacterium tuberculosis* at the concentration of 1 **µ**g/mL. INH is usually dosed at 5mg/kg/day, up to 300 mg/day, yielding a peak level in serum of 3–5 µg/ml [12]. Although INH-monoresistant TB can be successfully treated with at least 6-month Rifampin containing regimens, it is not clear whether this outcome is independent of the degree of high or low-concentration INH resistance. Since previous studies did not stratify the results according to low- and high-concentration INH resistance, outcomes for patients with high-concentration INH monoresistant TB may not be as good for patients with low-concentration INH monoresistant TB. The aim of this study was to evaluate the patients’ demographic characteristics, treatment regimens, and treatment outcomes for those with different concentrations of INH-monoresistant TB.

## Materials and Methods

### Study Population

We retrospectively recruited patients with culture-confirmed INH-monoresistant *Mycobacterium tuberculosis* during January 2006 to December 2007 in Chang Gung Memorial Hospital, a tertiary hospital in Taiwan. Patients were excluded if INH resistance was acquired during treatment or if resistance to any other first-line anti-TB medication was documented [4]. The Chang Gung Medical Foundation Institutional Review Board approved the study and waived the requirement for informed consent due to the retrospective nature of this study.

### Study Design

Each patient’s medical records were reviewed to collect the clinical characteristics and laboratory results. In addition, information including prior tuberculosis treatment, treatment regimens, adverse drug reactions, adherence to therapy, and clinical follow-up for 1 year after completion of treatment was analyzed.

### Definitions

Drug susceptibility was confirmed in all cases at the laboratory by the agar-proportion method [13]. INH resistance was classified as either low concentration or high concentration when there was **>**1% growth of *Mycobacterium tuberculosis* complex in the presence of 0.2 **µ**g/mL or 1 **µ**g/mL of INH, respectively [4]. A patient was defined as being cured if conversion from positive to negative sputum culture was achieved after the start of treatment and the patient remained culture-negative throughout the period of treatment. Sputum culture conversion was defined as the time in months from the time treatment was started to the time at which the first negative sputum culture was obtained. Treatment completion was defined as the patients who had completed treatment but did not meet the criteria to be classified as instances of cure or failure. In this study, both cure and treatment completion were regarded as treatment successes [9]. In accordance with the ATS/CDC/IDSA guidelines, a patient was considered to have treatment failure if culture results remained positive after 4 months of treatment, and if they had a relapse when a second episode of TB was diagnosed within 1 year after treatment completion [7]. The definition of default was a patient who missed >20% of their total doses or >2 months of consecutive therapy.

An adverse drug reaction was defined as any symptom or laboratory abnormality leading to an interruption of ≥1 dose of antituberculosis medication [4]. Patients were considered to have non-adherence to treatment if any of the following conditions were met: (1) more than 14 consecutive days of treatment were missed; (2) more than 2 consecutive visits to the clinic were missed; or (3) more than 20% of doses were missed in any month by a patient receiving directly observed therapy [4].

### Statistical Analysis

Data were expressed as mean ± SEM (standard error of the mean). The Student’s t test was used for comparisons of continuous variables between the two groups, while the Mann-Whitney test was used for non-normal distributions. Categorical variables were compared by **x**2 or Fisher’s exact tests. Univariable associations were reported and multivariable logistic regression was used to identify independent associations between measured covariates and the probability of unsuccessful treatment. The final multivariable model was constructed first by including all variables considered in the univariable analysis, then by sequentially removing explanatory variables with the greatest P value. If the effect size of the other explanatory variables changed by less than 10%, the variable was dropped from the model, otherwise it was retained. The log-rank test was used for analysis of the proportion of those remaining on therapy. A p value less than 0.05 was considered statistically significant. Analysis was carried out using SPSS (version 13.0; SPSS; Chicago, IL) statistical software.

## Results

### Demographic and Clinical Characteristics of Patients

A total of 1229 culture-positive tuberculosis patients were identified in our hospital from January 2006 to December 2007, of whom 134 (10.9%) had INH monoresistance and were included in this study. The baseline demographics and clinical characteristics of these patients are listed in Table 1. The mean ages of the patients with low- and high-concentration monoresistant TB were 53.2 and 58.8 years, respectively, with 77.3% and 73.3% males, respectively. Other characteristics including prior tuberculosis treatment, adherence to treatment and treatment duration were similar between the two groups.

**Table 1:** Demographic and clinical characteristics of the patients.

Characteristic	INH low concentration resistancen = 44	INH high concentration resistancen = 90	Odds ratio(95%CI)	p value
Male, n	34(77.3%)	66(73.3%)	1.24(0.53–2.88)	0.623
Age, years	53.2±3.7	58.8±3.0		0.264
Prior tuberculosis treatment	12(27.3%)	29(32.2%)	0.79(0.36–1.25)	0.559
Pulmonary tuberculosis	38(86.4%)	86(95.6%)	0.88(0.16–5.04)	0.889
Positive AFB smear test	30(68.2%)	60(66.7%)	1.50(0.65–3.47)	0.342
Cavitary chest radiograph	10(22.7%)	22(24.4%)	1.03(0.44–2.44)	0.946
Received initial isoniazid	42(95.5%)	88(97.8%)	0.48(0.06–3.51)	0.458
Directly observed therapy	44(100%)	85(93.3%)	5.73(0.31–106)	0.111
Adherence to treatment	43(95.5%)	87(96.7%)	1.48(0.15–14.69)	0.735
Adverse reaction	22(50%)	40(44.4%)	1.25(0.61–2.58)	0.545
Sputum culture conversion at ≤2 months	14(31.8%)	18(20%)	1.87(0.82–4.23)	0.132
Treatment duration, days	297.8±19.0	289.9±14.6		0.750

Abbreviations: INH: isoniazid; AFB: acid fast bacilli; CI: confidence interval.

Categorical data are expressed as number (%).

Continuous data are expressed as mean±SEM.

### Treatment Regimens for INH-monoresistant TB and the Reasons for Extension of Treatment Beyond 6 Months

The treatment regimens of the patients are shown in Table 2. Highly heterogeneous treatment regimens were used in both groups, and the treatment regimens and duration were similar between both groups. The most common regimen was 2 months of INH, rifampin, ethambutol, and pyrazinamide, followed by 5 to 7 months of rifampin, ethambutol and pyrazinamide treatment in both groups. Only a minor percentage of patients in both groups received 6-month treatment regimens (INH low-concentration resistance vs. high-concentration resistance, 9.1% vs. 13.3%, respectively). Table 3 lists the reasons for extension of treatment beyond 6 months in the patients with INH monoresistant TB. The most common reason for a treatment duration longer than 6 months was pyrazinamide (PZA) given for less than 6 months followed by a delay in clinical response to treatment. The reasons for extending treatment beyond 6 months was not significantly different between the two groups.

**Table 2:** Treatment regimens for isoniazid-monoresistant tuberculosis.

Treatment regimens	INH low concentrationresistance,n = 44	INH high concentrationresistance,n = 90	Odds ratio(95% CI)	p value
**6 Months, n(%)**	**4(9.1%)**	**12(13.3%)**	**0.65(0.2–2.15)**	**0.477**
HREZ (2), REZ (4)	4(9.1%)	10(11.1%)	0.80(0.24–2.71)	0.720
Other	0(0%)	2(2.2%)	0.4(0.02–8.47)	0.993
**7–12 Months**	**28(63.6%)**	**51(56.7%)**	**1.34(0.64–2.81)**	**0.441**
HREZ (2), REZ (5–7)	10(22.7%)	21(23.3%)	0.97(0.41–2.28)	0.938
HREZ (9)	5(11.4%)	10(11.1%)	1.03(0.33–3.21)	0.965
HRE (9–12)	3(6.8%)	4(4.4%)	1.57(0.34–7.36)	0.562
HREZ (2), RE (7–10)	4(9.1%)	2(2.2%)	2.90(0.62–13.57)	0.160
HREZ (2), REZ (7–10)	2(4.5%)	4(4.4%)	1.38(0.22–8.58)	0.728
HREZ (2), HRE (7–10)	2(4.5%)	4(4.4%)	1.02(0.18–5.82)	0.979
Other	2(4.5%)	6(6.7%)	0.38(0.08–1.82)	0.221
**>12 months**	**12(27.3%)**	**27(30%)**	**0.88(0.39–1.95)**	**0.744**
HREZ (2), RE (>10)	5(11.4%)	13(14.4%)	0.76(0.25–2.28)	0.623
HREZ (2), RZ (>10)	4(9.1%)	12(13.3%)	0.65(0.20–2.15)	0.477
Other	3(6.8%)	2(2.2%)	3.22(0.52–20.02)	0.187

Abbreviations: INH: isoniazid; CI: confidence interval.

E, ethambutol; H, isoniazid; R, rifampin; Z, pyrazinamide.

**Table 3:** Reasons for extension of treatment beyond 6 months.

Treatment regimens	INH low concentrationresistance, n = 44	INH high concentrationresistance, n = 90	Odds ratio(95% CI)	p value
Pyrazinamide given for <6 months, all	19(47.5%)	32(41.0%)	1.30(0.60–2.80)	0.502
Because of physician preference	3(7.5%)	6(7.7%)	0.97(0.23–4.11)	0.970
Because of adverse reaction	16(40.0%)	26(33.3%)	1.33(0.61–2.93)	0.474
Hepatotoxicity	8(20.0%)	8(10.3%)	2.19(0.75–6.35)	0.143
Hyperuricemia/gout	8(20.0%)	16(20.5%)	0.97(0.37–2.51)	0.948
Rash	0(0%)	2(2.6%)	0.38(0.02–8.06)	0.307
Treatment noncompliance	2(5.0%)	3(3.8%)	1.32(0.21–8.22)	0.768
Extrapulmonary tuberculosis	5(12.5%)	4(5.1%)	2.64(0.67–10.46)	0.153
Delayed clinical response to treatment	8(20.0%)	24(30.8%)	0.56(0.23–1.40)	0.213
Delayed culture conversion	6(15.0%)	15(19.2%)	0.74(0.26–2.09)	0.570

Abbreviations: INH: isoniazid; CI: confidence interval.

### Clinical Outcomes of Tuberculosis Patients with INH Monoresistant TB

**Figure 1:**
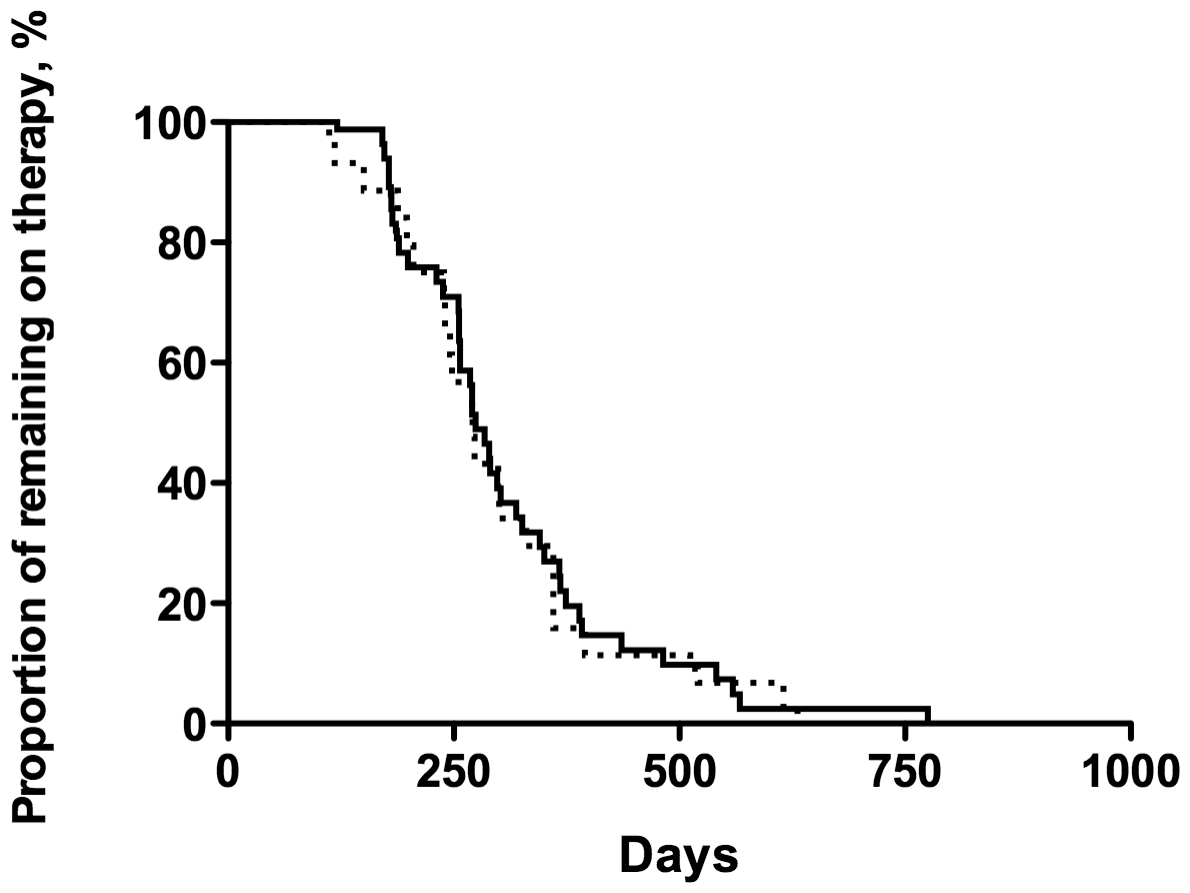
Kaplan-Meier analysis for patients with isoniazid (INH)-resistant tuberculosis (TB) remaining on treatment. Patients with low-concentration INH-monoresistant TB (dashed line) and high-concentration INH-monoresistant TB (solid line) received a similar duration of anti-TB therapy (p = 0.761 by the log-rank test, hazard ratio = 1.06, 95% confidence interval, 0.73–1.55).

The treatment success rates were high in the patients with INH monoresistant TB (Table 4), and the success rates between the patients with low- and high-concentration INH monoresistant TB were similar (81.8% vs. 86.7%, respectively). For the patients with unsuccessful treatment, death was the most common cause. According to the log-rank test (Figure 1), the proportion of those remaining on therapy was also similar between the two groups (p = 0.761, hazard ratio = 1.06, 95% confidence interval, 0.73–1.55).

**Table 4:** Clinical outcomes of the tuberculosis patients with INH monoresistance.

Treatment regimens	INH low concentration resistance, n = 44	INH high concentration resistance, n = 90	Odds ratio(95% CI)	p value
**Successful**	**36(81.8%)**	**78(86.7%)**		
Cure	35(79.5%)	75(83.3%)	0.78(0.31–1.95)	0.591
Completed	1(2.3%)	3(3.3%)	0.68(0.07–6.68)	0.735
**Unsuccessful**	**8(18.2%)**	**12(13.3%)**		
Default	1(2.3%)	2(2.2%)	2.07(0.13–33.91)	0.603
Failure	2(4.5%)	2(2.2%)	1.38(0.22–8.58)	0.728
Death	4(9.1%)	7(7.8%)	1.03(0.29–3.61)	0.969

Abbreviations: INH: isoniazid; CI: confidence interval.

In the study population, 28 patients received INH treatment for longer than 6 months while 106 patients received INH shorter than 6 months. In patients received INH treatment for longer than 6 months, their unsuccessful treatment rates were similar to that in patients received INH treatment shorter than 6 months (5/28, 17.9% vs. 15/106, 14.2%, Odds ratio, 1.32, 95% C.I., 0.43–4.01, p = 0.625). The unsuccessful treatment rates between the patients with low- and high-concentration INH monoresistant TB (2/10, 20% vs. 3/18, 16.7%, Odds ratio, 1.25, 95% C.I., 0.17–9.90, p = 0.825) were similar in those who have received INH treatment longer than 6 months.

There were 41 patients who had received prior anti-TB treatment while 93 patients were treatment naive TB. The unsuccessful treatment rates were higher in patients received prior TB treatment than those without prior TB treatment (10/41, 24.4% vs. 10/93, 10.8%, Odds ratio, 2.68, 95% C.I., 1.02–7.06, p = 0.041). The unsuccessful treatment rates between the patients with low- and high-concentration INH monoresistant TB (4/12, 33.3% vs. 6/29, 20.6%, Odds ratio, 1.92, 95% C.I., 0.43–8.59, p = 0.391) were similar in those who have received prior TB treatment.

### Multivariable Analysis

A multivariable analysis was performed to identify risk factors for unsuccessful treatment outcomes among patients with INH-monoresistant TB (table 5). In this multivariable analysis, prior tuberculosis treatment (Odds ratio, 2.82, 95% C.I., 1.02–7.77, p = 0.045) was the only independent risk factor for unsuccessful treatment outcome. INH high-concentration resistance was not an independent risk factor for unsuccessful treatment outcome in the multivariable analysis model.

**Table 5:** Univariable and multivariable associations with unsuccessful treatment outcome.

Variables	Univariate analysis		Multivariate analysis	
	Odds Ratio (95% C.I.)	p value	Odds Ratio (95% C.I.)	p value
Age>65 year-old	1.14(0.43–3.02)	0.788	1.24(0.42–3.64)	0.696
Prior tuberculosis treatment	2.68(1.02–3.05)	0.041	2.82(1.02–7.77)	0.045
Positive AFB smear test	1.17(0.42–3.28)	0.770	0.96(0.32–2.95)	0.947
Sputum culture conversion at ≤2 months	1.07(0.36–3.23)	0.899	0.96(0.29–3.18)	0.945
INH high-concentration resistance	0.69(0.26–1.84)	0.459	0.62(0.22–1.72)	0.357

Abbreviations: INH: isoniazid; AFB: acid fast bacilli; CI: confidence interval.

## Discussion

The findings of the present study demonstrated a high treatment success rate in patients with INH monoresistant TB in Taiwan. The unsuccessful treatment rates were higher in patients received prior TB treatment than those without prior TB treatment. In addition, different levels of INH monoresistance did not affect the treatment course and outcomes of these patients. In spite of highly heterogeneous treatment regimens, most of the patients with INH monoresistant TB received anti-TB therapy for a duration longer than 6 months. The prolonged treatment duration in these patients was mostly caused by PZA given less than 6 months and a delay in clinical response to treatment. Prior tuberculosis treatment was the only independent risk factor for unsuccessful treatment outcome in multivariable analysis.

The patients with low- and high-concentration INH monoresistant TB had similar demographic and clinical characteristics. In addition, the treatment outcomes, treatment regimens, and the reasons for extension of treatment beyond 6 months were not different between the two groups. Although originating from different gene mutations [10], [14], low- and high-concentration INH monoresistant TB had similar responses to therapy and clinical course, and therefore both groups should be treated aggressively. A multi-national study reported a treatment success rate of INH-resistant TB of 82% in new tuberculosis cases, compared to 54% in retreated TB cases [15]. In the present study, around 70% of the recruited patients were new tuberculosis cases. Thus, the treatment success rates were high in both the low- and high-concentration resistance groups.

Compatible with previous studies, the treatment success rate in this study was higher than 80% [10], [15]. One of the possible reasons for this is the high percentage (129/134, 96.3%) of directly observed therapy (DOT). The CDC of Taiwan has enforced the DOT program since 2006, and the implementation rate of DOT for TB case now exceeds 90% [16]. In Taiwan, once a TB case is verified, the patient is invited to participate in the DOT program. Among those who agree, an official DOT observer is assigned to the patient. The observer then monitors the ingestion of anti-TB medications, adverse events, and treatment complications during home visits and provides patients with food coupons as incentives. The DOT program has been documented to be effective in increasing the treatment success rate in TB patients [17]–[19]. The benefits of the DOT strategy are encouraging patients to continue treatment, and identifying those who miss treatment thereby preventing the occurrence of resistant strains. Another possible factor for the high success rate may be attributed to high patient compliance with the anti-TB therapy in the study subjects. In turn, the high treatment compliance rate may be associated with enforcement of the DOT program among these patients.

The Taiwan guidelines recommend a treatment regimen of 6 to 9 months of rifampin, pyrazinamide, and ethambutol, with or without INH [20]. Therefore, the most common treatment regimens in the study population were an initial combination of 4 drugs including INH, rifampin, ethambutol, and pyrazinamide for 2 months, followed by 5 to 7 months of rifampin, ethambutol, and pyrazinamide treatment. Since the bactericidal anti-TB drug, rifampicin, was used in the majority of patients in the study, the critical factor for treatment success in these patients may be the strong sterilizing ability of rifampicin [6]. In this study, adverse effects of the anti-TB drugs frequently developed in patients with INH-monoresistant TB. The high prevalence of drug-induced adverse effects contributed to PZA being used for less than 6 months in these patients. The patients subsequently received an extended period of anti-TB therapy. In addition, a delay in clinical response to treatment and delayed culture conversion also contributed to the prolonged treatment course in the patients with INH-monoresistant TB.

The study has demonstrated good clinical outcomes of patients with INH-monoresistant TB treated with long-course of Rifampin-containing regimens, regardless of the degree of resistance. However, there are two relevant questions unsolved. First, a prolonged course treatment of Rifampin containing regimens is the cornerstone for good outcomes in patients with INH-monoresistant TB and most of the patients in the study were treated accordingly. Therefore, the role of INH therapy in INH monoresistant TB patients received prolonged Rifampin containing treatment remains unresolved. Second, only 13% of patients with high-concentration INH monoresistant TB were successfully treated for 6 months. The 6-month treatment regimen was effective in minor population of those patients and the effects may be contributed from the combination of 4 anti-TB drugs.

The major limitations of the present study are its retrospective nature, which may have led to bias in patient selection. Second, the sample size of the study is small, and therefore the results of the study should be interpreted with caution. A prospective study with larger a sample size is warranted to further confirm the clinical outcomes in patients with different concentrations of INH-monoresistant TB. Third, the DOT program was commonly used in the study subjects. Therefore, the results of the study may only be applied to countries, which enforce a DOT program in the treatment of TB patients. Lastly, genetic analysis on the types of mutation was not performed in the study. The mutations in the katG and inhA gene are associated with high- and low- concentration INH resistance, respectively [10]. In Taiwan, a previous study has showed 48.5% of katG mutation, 30.3% of inhA, and 9.6% of katG and inhA double mutation in INH resistance [21]. The study investigating the mutation analysis and clinical outcome in INH monoresistant TB may provide important information.

The results of this study, if further confirmed by large-scaled studies, suggest that most of patients with INH-monoresistant TB can be successfully treated with prolonged Rifampin containing regimens. In addition, INH-monoresistant TB patients with history of prior tuberculosis treatment may have higher incidence of treatment failure. Those patients should receive aggressive treatment and closely monitoring of their response to anti-TB therapy.

In conclusion, different levels of INH resistance did not affect the treatment outcomes of patients with INH-monoresistant tuberculosis. In spite of relatively complicated treatment courses and heterogeneous treatment regimens, the patients with INH-monoresistant TB had a high treatment success rate. Prolonged Rifampin-containing regimens may achieve those good outcomes in patients with low- and high-concentration INH-monoresistant TB.
